# Anti-Apoptotic Effect of Tax: An NF-κB Path or a CREB Way?

**DOI:** 10.3390/v3071001

**Published:** 2011-06-27

**Authors:** Daniela Saggioro

**Affiliations:** Immunology and Molecular Oncology Unit, Veneto Institute of Oncology IOV-IRCCS, via Gattamelata 64, 35128 Padova, Italy; E-Mail: d.saggioro@unipd.it; Tel.: +39-0498215884; Fax: +39-0498072854

**Keywords:** HTLV-1, tax, apoptosis, NF-κB, PI3K/Akt, Ras, ERK, CREB

## Abstract

The NF-κB pathway is intimately linked to the survival of mammalian cells, and its activation by Tax has consequently been considered important for human T-cell leukemia/lymphoma virus type 1 (HTLV-1)-infected cell resistance to death. Very little emphasis has been given to other mechanisms, although Tax regulates the expression and activity of several cellular genes. The finding that CREB protein is activated in HTLV-1 infected cells underlines the possibility that other mechanisms of survival may be implicated in HTLV-1 infection. Indeed, CREB activation or overexpression plays a role in normal hematopoiesis, as well as in leukemia development, and CREB is considered as a survival factor in various cell systems. A better understanding of the different molecular mechanisms used by Tax to counteract cell death will also help in the development of new therapeutic strategies for HTLV-1 associated diseases.

## 1. Introduction

The human T-cell leukemia/lymphoma virus type 1 (HTLV-1) is the etiological agent of a highly aggressive and fatal disease called adult T-cell leukemia/lymphoma (ATLL) [1,2]. The virus is also the causative agent of tropical spastic paraparesis/HTLV-1-associated myelopathy (TSP/HAM), a degenerative neurological illness [3], and other diseases, including polymyositis, uveitis, infectious dermatitis, immunodeficiency and arthropathy [4]. The onset of these pathologies is believed to be mainly a consequence of the expression of the viral protein Tax, which is also considered the major oncogenic protein of HTLV-1. Indeed, Tax has been shown to induce leukemia in transgenic mice [5], and to immortalize human T lymphocytes when expressed in either a herpes- or a retroviral vector [6,7].

Tax is a 40 kDa phosphoprotein originally described as a nuclear protein [8,9], and subsequently found to shuttle from the nucleus to the cytoplasm [10–14]. In the nucleus, Tax is in part associated with speckled structures coincident with a subset of nuclear transcriptional hot spots [10], while in the cytoplasm Tax has been reported to be closely associated with Golgi compartments and localized in cell-cell contact regions [15]. The mechanisms regulating nucleus-cytoplasmic shuttling and targeting of Tax to distinct subcellular regions have yet to be determined, but it is conceivable that the pleiotropic nature of Tax activities might in part be determined by its subcellular localization.

The primary and most studied role of Tax is that of a transcriptional transactivator. Tax was identified as a *trans*-acting transcriptional activator for viral gene expression via the viral long terminal repeats (LTR) [16,17]. Successive studies have demonstrated that, by interacting with members of various transcription factor families that include cAMP-responsive element-binding protein/activating transcription factor (CREB/ATF), nuclear factor-κB (NF-κB), and serum responsive factor (SRF), Tax regulates not only the expression of HTLV-1, but also that of several cellular genes [17,18]. Furthermore, Tax has been found to modulate the function of numerous cellular proteins, including those involved in cell cycle regulation or belonging to signal transduction pathways and cytoskeleton, by directly interacting with them [19–23]. Tax expression has also been shown to reduce cellular genomic stability [24–27] and to interfere with most DNA repair mechanisms [28,29].

Although ATLL generally presents prolonged incubation periods and ultimately only a minor subset (2–5%) of infected individuals develop neoplasia, once the disease is diagnosed the prognosis is dismal. The poor outcome of patients with ATLL is mainly linked to intrinsic resistance of leukemic cells to conventional anticancer therapies that can be ascribed to decreased susceptibility to apoptosis shown by leukemic cells. Resistance to apoptosis is one of the hallmarks of malignant cell transformation [30] but also plays an important role in the pathogenesis of neurodegenerative and immunological disorders, all linked to HTLV-1 infection.

Apoptosis can occur via two principal routes: the extrinsic (receptor-mediated) pathway and the intrinsic (mitochondrial) pathway. In the receptor-mediated pathway, interaction of the receptor with its ligand results in the oligomerization of the receptor’s intracellular death domains, and activation of the initiator caspase-8. The intrinsic apoptotic pathway requires pro-apoptotic proteins of the Bcl-2 family which act principally at the mitochondrial level. Activation of these proteins by apoptotic signals leads to changes in mitochondrial outer membrane permeability, release of cytochrome c, and activation of the initiator caspase 9 through the formation of the apoptosome. Both pathways induce activation of executioner caspases, and subsequent controlled destruction of cells. The link between the receptor-mediated signaling cascade and the mitochondria is provided by the Bcl-2 family member Bid. Bid is cleaved by caspase-8 and, in its truncated form (tBID), translocates to the mitochondria where it acts in concert with the proapoptotic Bcl-2 family members Bax and Bak to induce the release of cytochrome c and other mitochondrial pro-apoptotic factors into the cytosol [31,32].

While unbalanced activation of signal transduction pathways, inhibition of cell cycle checkpoint, and accumulation of genetic defects are generally associated with cell transformation and escape from apoptosis, the contribution of Tax to apoptosis has been a matter of discussion. Tax has been found to either induce [33–42] or inhibit [43–51] apoptotic cell death triggered by stimuli that activate either the extrinsic or the intrinsic pathway. However, genome expression profiling of Tax-positive cells showed that the viral protein down modulates a wide range of pro-apoptotic factors and stimulates expression of factors acting as anti-apoptotic proteins [52,53]. At present, it is generally recognized that the anti-apoptotic activity of Tax overcomes its potential apoptotic role.

Although the mechanism involved in the anti-apoptotic effect of Tax remains to be defined, it is believed that Tax prevents apoptosis by interfering with cell survival signaling cascades. So far, much attention has been given to the NF-κB pathway, however, in the last years, at least two other cellular survival pathways have come to the forefront.

In this review we will briefly discuss new findings and our current understanding of how the NK-κB, PI3K/Akt, Ras/Raf/ERK pathways, and finally CREB activation, are engaged by Tax to overcome cell death.

## 2. NF-κB Pathway

NF-κB family proteins are expressed in the cytoplasm of virtually all cell types, where their activity is controlled by a family of regulatory proteins called inhibitors of NF-κB (IκB). NF-κB activation is tightly regulated by signals that degrade IκB. In the canonical NF-κB signaling pathway, IκB proteins are phosphorylated by an activated IκB kinase (IKK) complex. Phosphorylation leads to ubiquitination and degradation of IκB, thus leaving the p50-RelA/p65 complex free to migrate to the nucleus. The IKK complex is composed of the catalytic subunits IKKα and IKKβ and the regulatory subunit IKKγ, also known as NEMO (NF-κB essential modulator). The IKKβ component is essential for signaling via the canonical NF-κB pathway, while in the so-called non-canonical pathway IKKβ and IKKγ are dispensable and processing of NF-κB2/p100 to p52/RelB dimers involves IKKα homodimers. The canonical and non-canonical NF-κB pathways regulate different κB elements and, therefore, a distinct subset of NF-κB target genes are controlled by the two pathways [54].

**Figure 1:**
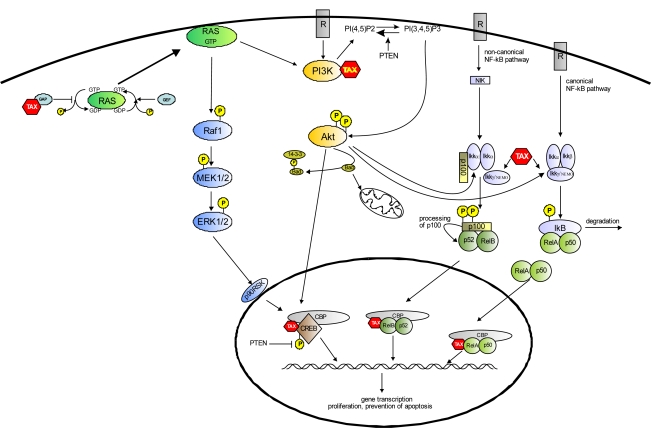
Survival pathways controlled by Tax. A growing body of evidence suggests that the anti-apoptotic effect of Tax is mediated by the activation of distinct signaling cascades, including NF-κB, PI3K/Akt and MEK/ERK1/2. Both canonical and non-canonical NF-κB pathways are activated by Tax, and control the expression of numerous survival genes; the PI3K/Akt pathway, also activated by Tax, acts by inhibiting the pro-apoptotic protein Bad, by activating the NF-κB pathway and inducing CREB phosphorylation; the Raf/MEK/ERK1/2 pathway is engaged by Tax through Ras activation. RasGTP can also induce PI3K activation; down modulation of PTEN expression by Tax leads to both Akt activation and increased levels of phosphorylated CREB in the nucleus.

In contrast to its transient mode of action during a physiological T-cell response, NF-κB is chronically activated in HTLV-1-transformed cell lines and freshly isolated ATLL cells [55], and this characteristic has been ascribed to Tax [56]. Tax interferes with the NF-κB pathway via direct Tax/IKKγ subunit interaction which leads to chronic IKK complex activation, continuous IκB degradation, and allows the translocation of NF-κB to the nucleus [57–61] (Figure 1). Another mechanism by which Tax contributes to NF-κB activation is the induction of the non-canonical pathway, leading to processing of p100 and the formation of p52/RelB complex. This process, that usually operates in B cells and lymphoid stromal cells [62], is very active in HTLV-1-transformed cells [63]. In contrast to the cellular pathway, Tax stimulated processing of p100 does not need NIK (NF-κB inducing kinase), but seems to require IKKγ [64,65] (Figure 1). Thus, whereas different physiological inducers of NF-κB activate either the canonical or non-canonical pathway, Tax can regulate both. Tax/NF-κB pathway interaction is not confined to the cytoplasm. Indeed, it has been reported that Tax can activate transcription by directly binding NF-κB subunits in the nucleus [66,67], and more recently, it has been shown that Tax sumoylation is critical for the recruitment of RelA to Tax nuclear bodies and transcriptional activation [68,69].

Activation of the NF-κB pathway is considered important for transformation, proliferation and survival of HTLV-1-infected cells. In accordance with this, treatment with specific NF-κB pathway inhibitors leads to suppression of growth and impaired tumorigenesis in mice of Tax-transformed fibroblasts [70], and induces apoptosis of HTLV-1-transformed T-cell lines and ATLL cells *in vitro* and *in vivo* [71–73].

## 3. PI3K/Akt Pathway

The PI3K/Akt signaling pathway is a key regulator of numerous physiological cellular processes, including proliferation and survival. Unrestrained activation of the PI3K/Akt pathway has been associated with malignant transformation and anti-apoptotic signaling.

The phosphatidylinositol 3-kinase (PI3K) is a heterodimer, composed of a catalytic subunit (p110) and a regulatory subunit (p85), which is activated through the interaction with tyrosine kinase receptors [74,75]. Akt, also known as protein kinase B (PKB), is a serine/threonine kinase and its activation is mediated by PI3K. Once activated, PI3K converts the plasma membrane lipid PI(4,5)P2 to PI(3,4,5)P3, and Akt is recruited by the latter to the plasma membrane. Translocation of Akt to the membrane and its interaction with PI(3,4,5)P3 is thought to provoke the exposure of two phosphorylation sites (Thr308 and Ser473); phosphorylation of Thr308 is mandatory for Akt activation while phosphorylation of Ser473 is required for full activation of the kinase [76]. Once activated Akt moves from the plasma membrane to both the cytoplasm and nucleus, where many of its substrates are located [76].

Akt regulates cellular survival by phosphorylation of substrates that directly or indirectly control the apoptotic machinery. For example, Akt induces the phosphorylation of Bad, a pro-apoptotic member of the Bcl-2 protein family; as a consequence, Bad dissociates from Bcl-X_L_ and associates with cytoplasmic 14-3-3 proteins with consequent loss of apoptotic activity [77]. Akt also appears to both negatively regulate factors that promote the expression of apoptotic genes and positively regulate factors that induce survival genes. An example is Akt’s ability to activate the canonical and the non-canonical NF-κB pathway by triggering IκB phosphorylation and degradation, and by promoting the processing of p100 to p52, respectively [78,79]. Besides the NF-κB pathway, Akt phosphorylates and activates CREB mediated transcription, thus controlling expression of numerous “survival” genes [80,81] (Figure 1).

Negative regulation of the PI3K/Akt pathway is mainly accomplished by the tumor suppressor PTEN (phosphatase and tensin homologue deleted on chromosome 10), through its dual function as lipid and protein phosphatase, and by SHIP (src homology 2 domain containing inositol polyphosphate phosphatase-1). They regulate intracellular levels of activated Akt by dephosphorylating PI(3,4,5)P3; thus, loss of PTEN or SHIP expression leads to permanent activation of the PI3K/Akt signaling pathway [82,83].

Akt has been found to be activated in HTLV-1-transformed cells [84–86], and its activation has been linked to apoptotic resistance. Peloponese *et al.* [87] suggested that Tax promotes Akt phosphorylation by directly binding the p85 subunit of PI3K, and that, in the absence of NF-κB activation, Akt can promote survival through activation of AP-1 (activator protein-1). Ikezoe *et al.* [82] reported that downstream of Akt, mTor (mammalian target of rapamycin) was activated in HTLV-1-infected cells and that treatment with rapamycin (the inhibitor of mTor) surprisingly led to phosphorylation of Akt at Ser473. More recently, it has been suggested that Tax activates the PI3K signaling cascade by down regulating the PI(3,4,5)P3 phosphatases PTEN and SHIP-1 [88].

Consistent with the premise that Akt is one of the survival mechanism of Tax, treatment of Tax-positive cells with PI3K/Akt pathway inhibitors induces cell death [85,89].

## 4. CREB Activation

CREB is a ubiquitously expressed, phosphorylation-dependent, transcriptional factor which acts by binding to cAMP response element (CRE) consensus sequence, as a homodimer or by forming a heterodimer with other members of the CREB family. Numerous stimuli and, by consequence, several kinases including Akt, p90rsk, protein kinase A and calcium/calmodulin-dependent kinases can phosphorylate CREB [90]. Although different residues may control CREB-dependent transcription, phosphorylation at Ser133 is essential for its activation by favoring CREB association with the histone acetyl-transferase paralogs CBP (CREB-binding protein)/p300, and subsequent regulation of a multitude of genes. Indeed, consensus CRE sequences, or slight variants of this sequence, have been identified in hundreds of cellular genes. More recently, a phosphorylation-independent transcriptional activity of CREB, via its interaction with the transducers of regulated CREB activity (TORCs), has been reported. TORC recruitment does not seem to improve CREB/DNA binding, but rather it enhances the interaction of CREB with a component of the transcriptional factor TFIID [91].

The vast number of functionally different genes regulated by CREB point out its critical relevance for many physiological cellular processes, including cell growth, and immune response [90,92–95], or aberrant processes as escape from apoptosis and cell transformation [96–101]. Indeed, microarray analysis of cells treated with CRE decoy oligodeoxynucleotide revealed that many genes related to tumor growth are regulated by the CREB family of transcription factors [102,103].

The role of CREB in HTLV-1-infected cells has thus far been considered only in terms of viral LTR activation. Transcription driven by cellular CREs, which lack the required GC-rich flanking sequences present in the viral CRE, have been considered less affected by Tax. However, the recent discovery that Tax might directly interact with the CREB co-activator TORC family of proteins, has uncovered the possibility that transcription of a significant number of cellular genes containing CRE sequences may be deregulated by Tax [104–107].

Our studies on the anti-apoptotic effects of Tax have indicated that CREB activation, rather than its NF-κB transcriptional activity, is important in preventing cell death [47,49,108]. Indeed, we have shown that induction of a specific block in CREB transactivation using dominant negative CREB mutants increased apoptosis, whilst triggering CREB phosphorylation with forskolin reduced apoptosis [108]. We have also observed that HeLa cells expressing Tax exhibit higher levels of Ser133-phosphorylated CREB compared to control cells, suggesting that Tax might influence the phosphorylation state of CREB [49].

In agreement with our results, Kim *et al.* [109] observed higher levels of intracellular p-CREB in a panel of HTLV-1-infected *versus* uninfected T cell lines. They also demonstrated that Tax expression was directly involved in the enhanced CREB phosphorylation. These findings suggest that the virus has evolved a mechanism to elevate pCREB levels in the HTLV-1-infected cells, likely as a way to promote strong Tax-mediated transactivation of CREB-responsive genes. It also seems that in HTLV-1-infected cells [109] or in HeLa cells transfected with Tax [108], the intracellular CREB is maximally phosphorylated, when compared to forskolin treatment.

Interestingly, Wu *et al.* [23], using a proteomic approach, reported that Tax can bind to several small GTPase-cytoskeleton proteins, including RhoA, Rac, Cdc42, and the RasGTPase activating protein GAP^1m^. In addition, using HeLa cells, we showed that while Tax physically interacts with GAP^1m^, its CREB-deficient mutant M47 (unable to protect cells from apoptosis [108]), binds to it with lower affinity [110]. Based on these findings, we proposed a model of Tax-mediated RasGTP (active form) accumulation, Raf/MEK/ERK pathway activation and CREB phosphorylation. In line with this, it has been shown that the inhibition of protein geranylgeranylation or farnesylation has anti-proliferative and apoptotic effects in HTLV-1-infected cells [111].

CREB is also a target of Akt and, as mentioned above, Tax can activate the PI3K/Akt pathway both by interacting with PI3K or down regulating the expression of PTEN [88]; it is also interesting to point out that PI3K is a downstream effector of Ras. In addition, more recently, Gu *et al.* [112] reported that PTEN phosphatase activity is required for CREB dephosphorylation at Ser133 in the nucleus, suggesting that PTEN deficiency (or down regulation) can increase the levels of CREB phosphorylation independently of PI3K/Akt or Raf/MEK/ERK activity (Figure 1).

## 5. Conclusions

The Tax protein is considered the main oncogenic product of HTLV-1; most likely one of the mechanisms involved in Tax-mediated transformation is its capability to alter the delicate balance between cell death and survival. Much evidence has led to consider the activation of the NF-κB pathway as the principal survival mechanism of Tax, and has restricted Tax-induced CREB activity to viral gene expression. However, the information gathered so far suggests that Tax, besides activation of the NF-κB pathway, can exert its anti-apoptotic activity by affecting CREB phosphorylation through activation of the PI3K/Akt and, possibly, of the Raf/MEK/ERK pathways, both of which list CREB as a downstream effector.

Thus, in order to be more effective, therapeutic approaches to ATLL must take into account the many interconnections between the survival pathways engaged by Tax, and develop strategies that simultaneously block different targets.
